# Breast cancer outcome in relation to bone mineral density and bisphosphonate use: a sub-study of the DATA trial

**DOI:** 10.1007/s10549-020-05567-9

**Published:** 2020-03-02

**Authors:** Irene E. G. van Hellemond, Carolien H. Smorenburg, Petronella G. M. Peer, Astrid C. P. Swinkels, Caroline M. Seynaeve, Maurice J. C. van der Sangen, Judith R. Kroep, Hiltje de Graaf, Aafke H. Honkoop, Frans L. G. Erdkamp, Franchette W. P. J. van den Berkmortel, Wilfred K. de Roos, Sabine C. Linn, Alexander L. T. Imholz, Maaike de Boer, Vivianne C. G. Tjan-Heijnen, the Dutch Breast Cancer Research Group (BOOG)

**Affiliations:** 1 Department of Medical Oncology, GROW – School for Oncology and Developmental Biology, Maastricht University Medical Centre, Maastricht, The Netherlands; 2 Department of Internal Medicine, Medical Centre Alkmaar, Alkmaar, The Netherlands; 3 Biostatistics, Radboud Institute for Health Sciences, Radboud University Medical Centre, Nijmegen, The Netherlands; 4 Clinical Research Department, Netherlands Comprehensive Cancer Organization IKNL, Utrecht, The Netherlands; 5 Department of Medical Oncology, Erasmus MC Cancer Institute, Rotterdam, The Netherlands; 6 Department of Radiation Oncology, Catharina Hospital, Eindhoven, The Netherlands; 7 Department of Medical Oncology, Leiden University Medical Centre, Leiden, The Netherlands; 8 Department of Medical Oncology, Medical Centre Leeuwarden, Leeuwarden, The Netherlands; 9 Department of Medical Oncology, Isala Clinics, Zwolle, The Netherlands; 10 Department of Medical Oncology, Zuyderland Medical Centre, Sittard, The Netherlands; 11 Department of Medical Oncology, Zuyderland Medical Centre, Heerlen, The Netherlands; 12 Department of Surgery, Gelderse Vallei Hospital, Ede, The Netherlands; 13 Department of Medical Oncology, Netherlands Cancer Institute, Amsterdam, The Netherlands; 14 Department of Medical Oncology, Deventer Hospital, Deventer, The Netherlands; 15 Division of Medical Oncology, Department of Internal Medicine, Maastricht University Medical Centre, P.O. Box 5800, 6202 AZ Maastricht, The Netherlands

**Keywords:** Breast cancer, Tamoxifen, Aromatase inhibitor, Bone health, Osteoporosis, Bisphosphonates, Survival, Bone metastases, Distant recurrence-free survival

## Abstract

**Purpose:**

The phase III DATA study compared 6 and 3 years of adjuvant anastrozole following 2–3 years of tamoxifen in postmenopausal breast cancer patients. This pre-planned side-study assessed the relationship between a reduced bone mineral density (BMD) and distant recurrence-free survival (DRFS), and evaluated the effect of bisphosphonates on DRFS.

**Methods:**

We selected all patients with a BMD measurement within 3 years after randomisation (landmark) without any DRFS events. Kaplan–Meier methods and Cox proportional hazards models were used for analyses.

**Results:**

Of 1860 eligible patients, 1142 had a DEXA scan before the landmark. The BMD was normal in 436 (38.2%) and showed osteopenia in 565 (49.5%) and osteoporosis in 141 (12.3%) patients. After a median follow-up of 5.0 years from the landmark, neither osteopenia nor osteoporosis (compared with normal BMD) were associated with DRFS in both the 6-year [osteopenia HR 0.82 (95% CI 0.45–1.49), osteoporosis HR 1.10 (95% CI 0.26–4.67)] and the 3-year arm [osteopenia HR 0.75 (95% CI 0.40–1.42), osteoporosis HR 1.86 (95% CI 0.43–8.01)]. Moreover, bisphosphonate use did not impact DRFS.

**Conclusion:**

No association was observed between a reduced BMD and DRFS. Neither did we observe an impact of bisphosphonates on DRFS.

**Electronic supplementary material:**

The online version of this article (10.1007/s10549-020-05567-9) contains supplementary material, which is available to authorized users.

## Introduction

Bisphosphonates, in addition to supplementation of vitamin D and calcium, are pivotal in the medical treatment of osteoporosis. Aside from preventing bone loss and fractures, the use of bisphosphonates in the adjuvant setting has been shown to improve breast cancer outcome in postmenopausal breast cancer patients [1–6]. The Early Breast Cancer Trialists’ Collaborative Group (EBCTCG) meta-analysis observed a significant improvement in the rate of distant recurrences (18.4% in the bisphosphonate group versus 21.9% in the group without bisphosphonate, *p* < 0.001), mainly driven by a reduction in bone recurrences (5.9% versus 8.8%, respectively, *p* < 0.001), and a lower 10-year breast cancer mortality [7]. The effect was seen irrespective of bisphosphonate type. Additionally, in epidemiological studies bisphosphonate use for osteoporosis in healthy postmenopausal women was associated with a 30% reduced risk of breast and colon cancer [8]. Moreover, neoadjuvant use of bisphosphonates in combination with chemotherapy in women with stage II/III breast cancer resulted in an improved clinical and pathological response rate in postmenopausal women only [9]. Nevertheless, it remains insufficiently clear how to explain the effects of bisphosphonates on breast cancer prevention and recurrence, respectively. Is the effect directly caused by the bisphosphonates? Or are women with early breast cancer and osteoporosis simply at a lower risk of developing metastases due to lower oestrogen levels than in those without osteoporosis? Also, earlier studies showed that a reduced bone mineral density (BMD) was associated with a lower risk of breast cancer [10, 11].

The phase III DATA trial investigated the efficacy of 6 versus 3 years of anastrozole after an initial 2–3 years of tamoxifen in postmenopausal women with early breast cancer. Earlier, we reported on patterns of care considering bone health in these women, and the trend of BMD over time, and the incidence of fractures during and after cessation of anastrozole treatment [12]. In the current pre-planned side-study we assessed the relationship between a reduced BMD and late distant recurrence-free survival (DRFS) (more than 5 years after breast cancer diagnosis), and evaluated the effect of bisphosphonates on late DRFS.

## Methods

### Study design, participants and procedures

The DATA trial included 1860 eligible postmenopausal women with hormone receptor-positive early breast cancer who had already received 2–3 years of adjuvant tamoxifen after curative local treatment, and who were without signs of loco-regional and/or distant metastases. Patients used anastrozole for 6 or 3 years according to randomisation. Ethics approval was obtained at the central commission of research involving humans in Nijmegen in the Netherlands. The DATA trial (NCT00301457) is described in detail elsewhere [13]. Decisions on BMD measurements and bisphosphonate use were left to the treating physician. The DATA study protocol advised to follow the recommendations of (inter)national guidelines. During the conduct of the study adjuvant bisphosphonates were not recommended, therefore they were predominantly prescribed as treatment for osteopenia and osteoporosis (*T*-score ≦ − 2.0). We registered all BMD measurements and start of bisphosphonate use. BMD was measured by a dual-energy x-ray absorptiometry (DEXA) scan of the lumbar spine/hip. For the current analyses, DATA patients were selected who had a DEXA scan within 3 years after randomisation and did not have any distant recurrences or death (flow chart shown in Supplemental Fig. 1). Of the 1860 patients who were eligible for the DATA trial, 1714 patients had not experienced a DRFS event before the 3-year landmark. Amongst them, 1142 had at least one BMD measurement before the 3-year landmark.

### Statistical analysis

We registered all results of DEXA scans performed within three years after randomisation. The outcomes (*T*-scores) were categorised according to the world health organization classification for BMD; normal BMD *T*-score ≥ − 1.0 standard deviation (SD), osteopenia *T*-score < − 1.0 and > − 2.5 SD, and osteoporosis *T*-score ≤ − 2.5 SD [14]. Assessment of osteopenia and osteoporosis was based on the lowest available *T*-score in either the hip or the lumbar spine. Based on the result of the DEXA scan we classified the patients in three groups (normal BMD, osteopenia, and osteoporosis). The landmark method was used to assess the survival after a particular point in time. The DRFS time was measured from the landmark of 3 years after randomisation to distant recurrence or death, the so-called residual survival, and was censored at the date of last follow-up. DRFS rates were obtained with the Kaplan–Meier method. We analysed the relationship between BMD and DRFS by comparing women having either osteopenia or osteoporosis with those having a normal BMD in a Cox proportional hazards model. Secondly, we performed the same analyses selecting only those patients who had not received bisphosphonates before the landmark of 3 years, thereby correcting for a potential effect of bisphosphonates on DRFS. The hazard ratios (HR) were adjusted for tumour status, nodal status, tumour grade, and hormone receptor status. Further, we evaluated the effect of bisphosphonates, started before the 3-year landmark for a reduced BMD, on late DRFS by comparing the women with and without bisphosphonates. We used the term late DRFS since these recurrences occurred more than 5 years after breast cancer diagnosis. All reported *P* values are two-sided and a *p* value ≤ 0.05 was considered statistically significant. All analyses were performed using SAS version 9.2.

## Results

Of the 1860 randomised eligible DATA patients, 1142 (65.5% in the 6-year arm and 62.9% in the 3-year arm) had a DEXA scan within the first 3 years after randomisation. The median age at randomisation was 57.5 years (interquartile range 51.0–63.0), 67.3% of the patients had node-positive disease and 71.3% underwent (neo-)adjuvant chemotherapy. Except for T-stage, the baseline characteristics were well balanced between the BMD groups (Table 1). Women with a normal BMD more frequently had a larger tumour size at diagnosis, but the patient characteristics were similar to these of the total study population (Supplemental Table 1).

**Table 1:** Baseline characteristics of all eligible randomised patients in the DATA study who underwent a DEXA scan before the landmark of 3 years after randomisation

Characteristic	Total group (*n* = 1142)	Normal BMD (*n* = 436)	Osteopenia (*n* = 565)	Osteoporosis (*n* = 141)
Age at randomisation—no. (%)				
Median age at randomisation (IQR)	57.5 (51.0–63.0)	56.8 (51.0–62.0)	57.6 (51.0–64.0)	59.0 (51.0–64.0)
< 49 years	227 (19.9)	94 (21.6)	113 (20.0)	20 (14.8)
50–59 years	462 (40.5)	175 (40.1)	227 (40.2)	60 (42.6)
≥ 60 years	453 (39.7)	167 (38.3)	225 (39.8)	61 (43.3)
Tumour status—no. (%)				
pT1	519 (45.5)	170 (39.0)	276 (48.9)	73 (51.8)
pT2	540 (47.3)	237 (54.4)	244 (43.3)	59 (41.8)
pT3/4	82 (7.2)	29 (6.7)	44 (7.8)	9 (6.4)
Unknown	1	0	1	0
Nodal status—no. (%)				
pN0/pN0(i +)	373 (32.7)	136 (31.2)	190 (33.6)	47 (33.3)
pN1	612 (53.6)	235 (53.9)	300 (53.1)	77 (54.6)
pN2/pN3	157 (13.8)	65 (14.9)	75 (13.3)	17 (12.1)
Histological grade—no. (%)				
Grade I	202 (18.2)	72 (16.9)	103 (18.7)	27 (20.0)
Grade II	571 (51.4)	226 (53.1)	277 (50.4)	68 (50.4)
Grade III	338 (30.4)	128 (30.0)	170 (30.9)	40 (29.6)
Unknown	31	10	15	6
Hormone receptor status—no. (%)				
ER and PR positive	877 (76.8)	346 (79.4)	428 (75.8)	103 (73.1)
ER or PR positive	265 (23.2)	90 (20.6)	137 (24.2)	38 (26.9)
HER2 status—no. (%)				
Positive	19 (1.8)	7 (1.7)	11 (2.1)	1 (0.7)
Negative	1063 (98.2)	406 (98.3)	523 (97.9)	134 (99.3)
Unknown	60	23	31	6
Histology—no. (%)				
Lobular	207 (18.1)	80 (18.4)	100 (17.7)	27 (19.2)
Other	935 (81.9)	356 (81.7)	465 (82.3)	114 (80.9)
Type of breast surgery—no. (%)				
Breast-conserving surgery	568 (49.7)	214 (49.1)	291 (51.5)	63 (44.7)
Mastectomy	574 (50.3)	222 (50.9)	274 (48.5)	78 (55.3)
Type of axillary surgery—no. (%)				
Sentinel node only	310 (27.1)	129 (29.6)	144 (25.5)	37 (26.2)
Axillary lymph node dissection only	298 (26.1)	106 (24.3)	158 (28.0)	34 (24.1)
Sentinel node plus axillary lymph node dissection	517 (45.3)	195 (44.7)	253 (44.8)	69 (48.9)
None	17 (1.5)	6 (1.4)	10 (1.7)	1 (0.7)
Radiotherapy—no. (%)				
Local	320 (28.0)	119 (27.3)	162 (28.6)	39 (27.7)
Regional lymph nodes	24 (2.1)	12 (2.8)	9 (1.6)	3 (2.1)
Local and regional lymph nodes	414 (36.3)	159 (36.5)	201 (37.2)	45 (31.9)
None/unknown	384 (33.6)	146 (33.5)	184 (32.6)	54 (38.3)
Prior (neo)adjuvant chemotherapy—no. (%)^a^				
Anthracycline- and taxane-containing regimen	77 (6.7)	25 (5.7)	39 (6.9)	13 (9.2)
Anthracycline-containing regimen without taxane	712 (62.3)	281 (64.4)	350 (61.9)	81 (57.4)
Taxane-containing regimen without anthracycline	6 (0.5)	1 (0.2)	4 (0.7)	1 (0.7)
Regimen without anthracycline or taxane	19 (1.7)	6 (1.4)	13 (2.3)	0 (0.0)
No chemotherapy	328 (28.7)	123 (28.2)	159 (10.4)	46 (32.6)
Prior HER2-targeted therapy—no. (%)				
Yes	3 (0.4)	3 (1.0)	0 (0.0)	0 (0.0)
Previous duration of tamoxifen				
Median and IQR (years)	2.3 (2.1–2.5)	2.3 (2.1–2.5)	2.3 (2.1–2.5)	2.3 (2.1–2.8)
Treatment with bone protecting agents at inclusion—no. (%)				
Bisphosphonates	139 (12.2)	5 (1.2)	73 (12.9)	61 (43.3)
Vitamin D and/or Calcium	375 (32.8)	83 (19.0)	216 (38.2)	76 (53.9)

*TX* size of tumour could not be assessed, *ER*: oestrogen receptor, *PR* progesterone receptor, *HER2* human epidermal growth factor receptor 2

^a^All patients received cyclophosphamide-based chemotherapy

**Fig. 1:**
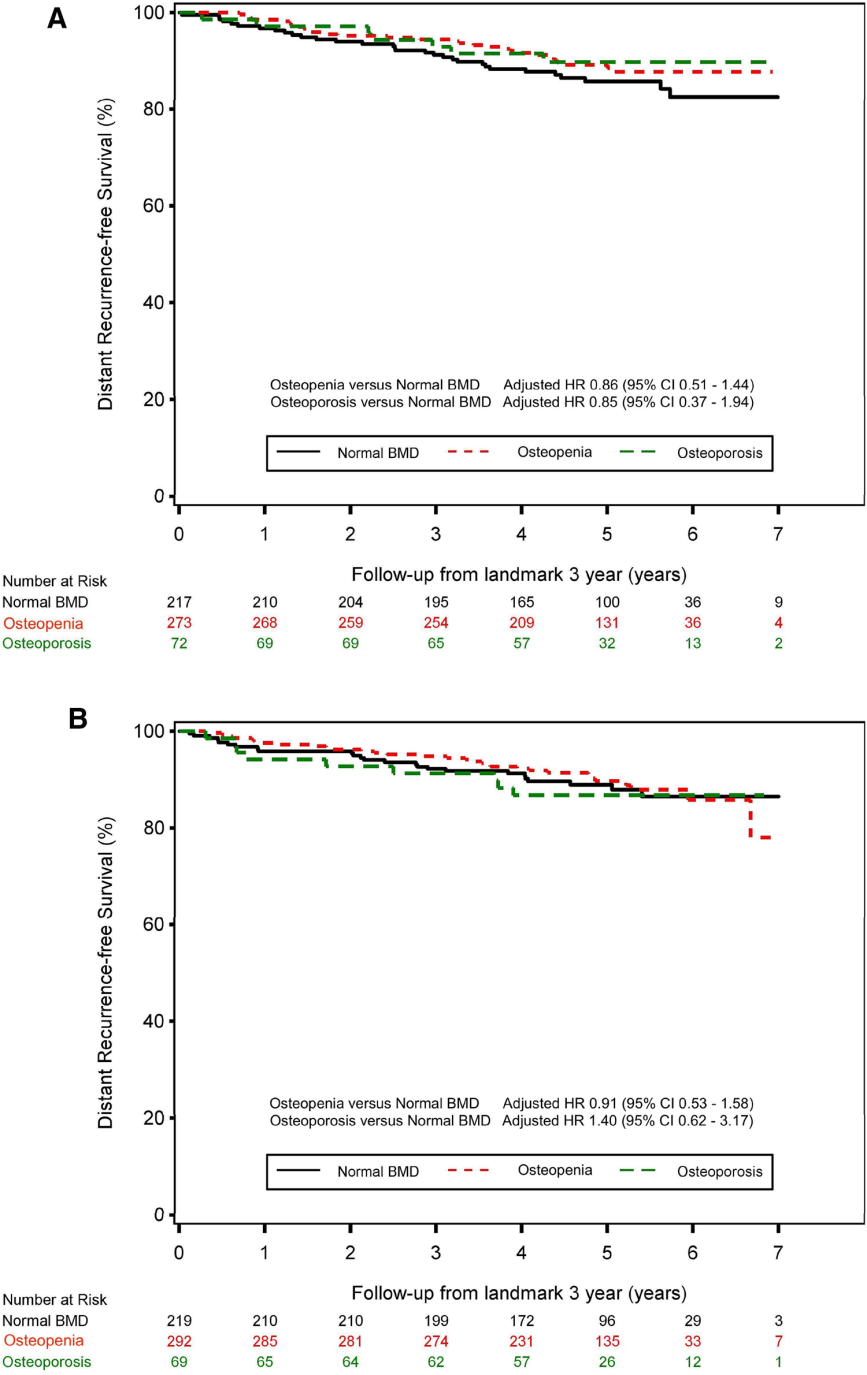
The impact of BMD on distant recurrence-free survival for the patients in **a** the 3-year anastrozole treatment arm, **b** the 6-year anastrozole treatment arm. Hazard ratios were adjusted for tumour size, nodal status, tumour grade and hormone receptor status

At the 3-year landmark, the BMD was considered normal in 436 (38.2%), showed osteopenia in 565 (49.5%), and osteoporosis in 141 (12.3%) patients. Seventeen (3.4%) patients of the normal BMD group used bisphosphonates in comparison with 161 (28.5%) in the osteopenia group, and 112 (80.9%) in the osteoporosis group. The median follow-up from the landmark was 5.0 years (interquartile range 4.3 to 5.7). The number of DRFS events were 61 and 66 in the 6- and 3-year arm, respectively. In the 6-year arm the 5-year residual DRFS rate was 89.7% in the osteopenia group, 86.7% in the osteoporosis group, and 88.9% in the normal BMD group [osteopenia versus normal BMD: adjusted HR 0.91 (95% CI 0.53–1.58); osteoporosis versus normal BMD: adjusted HR 1.40 (95% CI 0.62–3.17)]. In the 3-year treatment arm the 5-year residual DRFS rate was 89.2% in the osteopenia group, 89.7% in the osteoporosis group and 85.8% in the normal BMD group [osteopenia versus normal BMD: adjusted HR 0.86 (95% CI 0.51–1.44); osteoporosis versus normal BMD: adjusted HR 0.85 (95% CI 0.37–1.94)] (Fig. 1a, b).

**Fig. 2:**
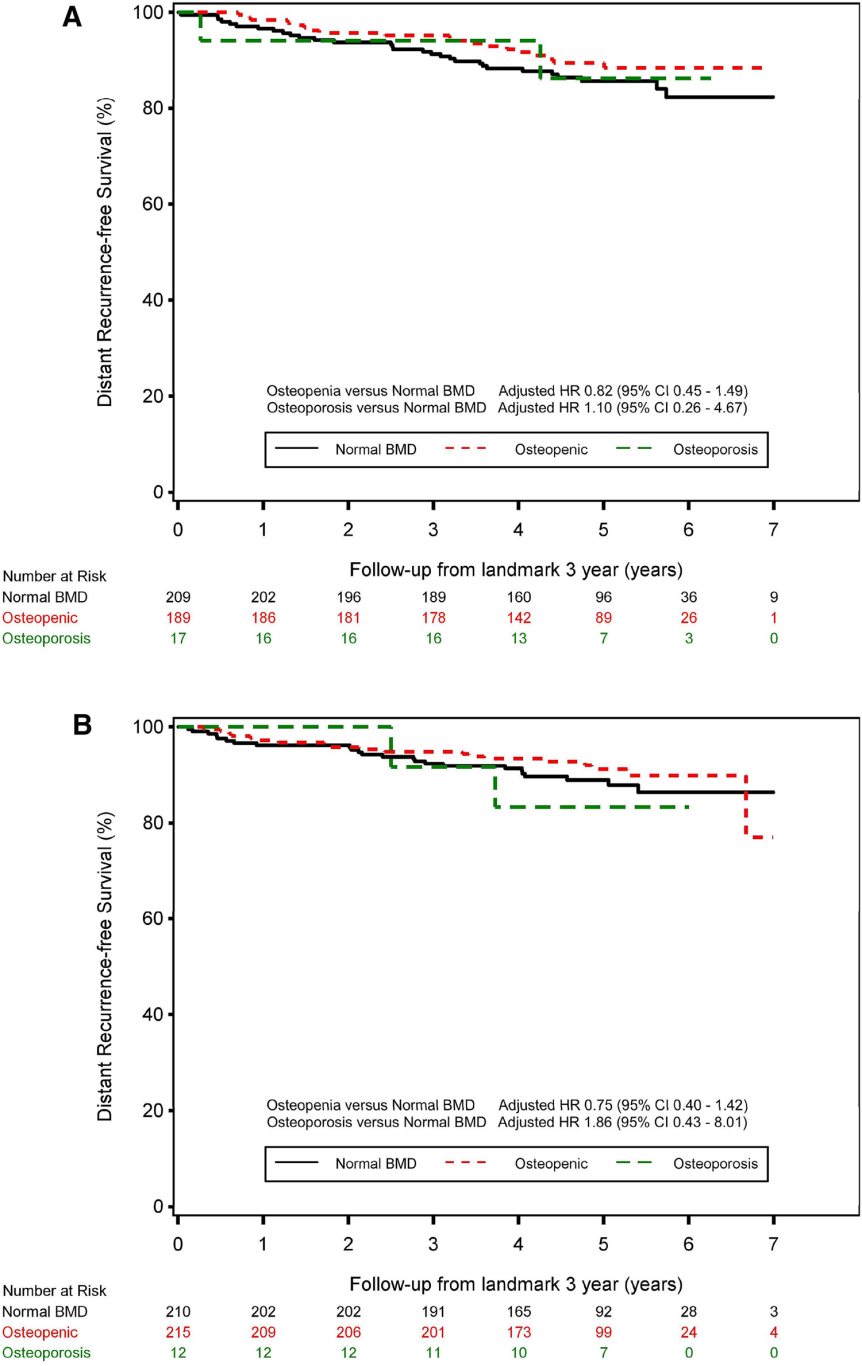
The impact of BMD on late DRFS selecting only the patients without bisphosphonates before the landmark in **a** the 6-year anastrozole treatment arm, **b** the 3-year anastrozole treatment arm. Hazard ratios were adjusted for tumour size, nodal status, tumour grade and hormone receptor status

When we repeated the analyses selecting only those patients who did not use bisphosphonates (*n* = 852) we neither observed an impact of BMD on late DRFS [6-year arm osteopenia versus normal BMD: adjusted HR 0.82 (95% CI 0.45–1.49); osteoporosis versus normal BMD: adjusted HR 1.10 (95% CI 0.26–4.67); 3-year arm osteopenia versus normal BMD: adjusted HR 0.75 (95% CI 0.40–1.42); osteoporosis versus normal BMD: adjusted HR 1.86 (95% CI 0.43–8.01)] (Fig. 2a, b). The number of 5-year DRFS events were 42 and 50 in the 6- and 3-year arm, respectively.

**Fig. 3:**
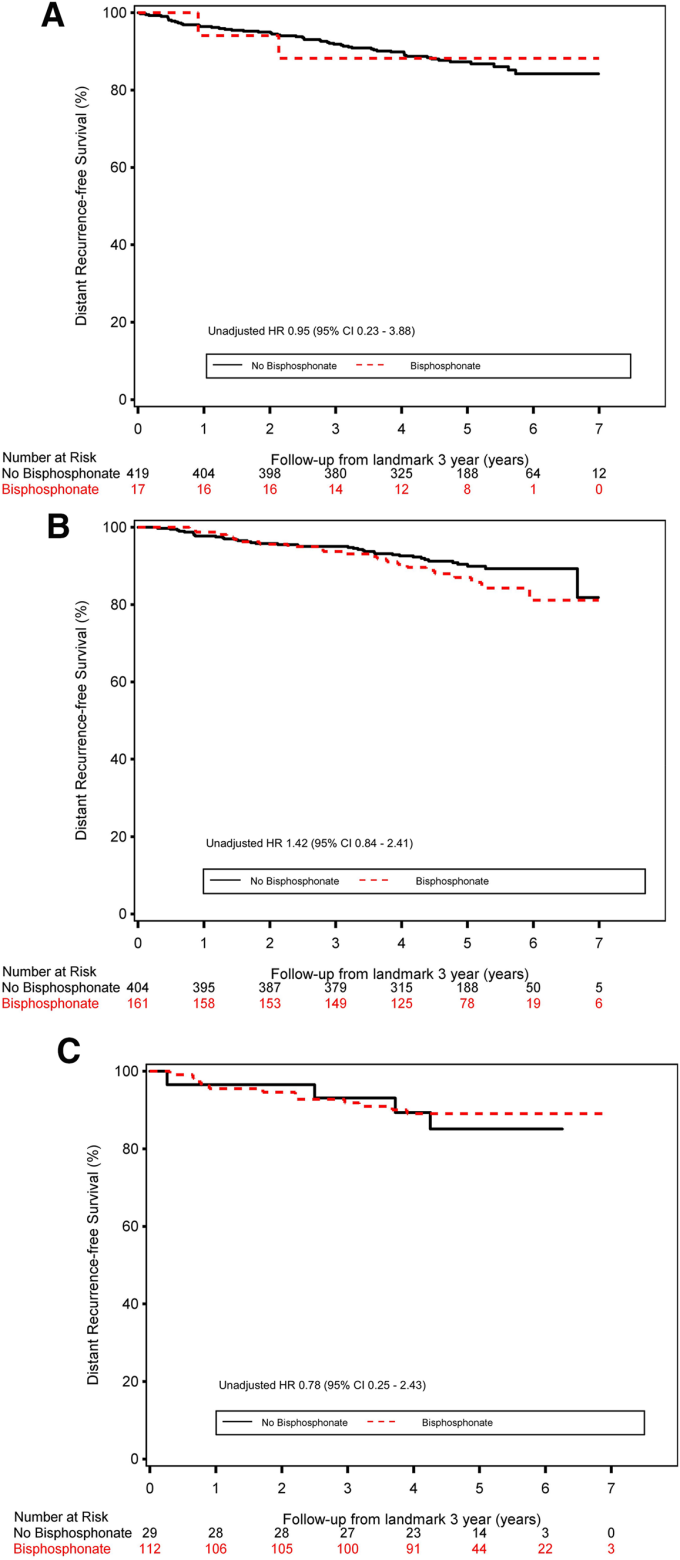
The impact of bisphosphonate use before the landmark on late DRFS in the women with A) a normal BMD, B) osteopenia, and C) osteoporosis at the 3-year landmark

Bisphosphonate treatment was started at a median *T*-score of − 2.3 (IQR − 2.7 to − 1.7). Of the patients in whom bisphosphonates were prescribed, the majority used oral bisphosphonates (59.3% alendronate, 27.0% risedronate, 0.6% clodronate, 6.6% ibandronate) and few used intravenous bisphosphonates (3.2% pamidronate, 2.7% zoledronate) [12]. Only 0.6% received denosumab. After a median follow-up of 5.0 years, the use of bisphosphonates before the landmark did not lead to a better DRFS in each of the BMD categories in comparison with women without bisphosphonates, normal BMD unadjusted HR − 0.95 (95% CI 0.23–3.88), osteopenia unadjusted HR 1.42 (95% CI 0.84–2.41), and osteoporosis unadjusted HR 0.78 (95% CI 0.25–2.43) (Fig. 3a–c).

## Discussion

The phase III DATA study investigates the optimal duration of adjuvant anastrozole (6 versus 3 years) in postmenopausal women with hormone receptor-positive breast cancer, after previous 2–3 years of adjuvant tamoxifen. In this pre-planned side-study we found no relationship between BMD and late DRFS (more than 5 years after diagnosis). Moreover, we did not observe a relationship between bisphosphonate treatment, predominantly started for a reduced BMD, and late DRFS.

Multiple observational trials observed that a reduced BMD was associated with a lower risk of developing breast cancer [10, 11], but it is insufficiently clear whether a reduced BMD in early breast cancer patients is related to a lower risk of distant recurrences. We were not able to show such a relationship in DATA patients, neither after adjusting for bisphosphonate use, as this could be a potential confounder. To our knowledge, this has only been studied (partly) in four studies. First, in the MA.14 trial it was studied whether baseline beta C-telopeptide, a marker of bone resorption, predicted relapse in postmenopausal breast cancer patients [15]. They showed that a higher bone resorption was associated with a higher incidence of bone metastases [HR 2.80 (95% CI 1.05–7.48; *p* 0.03)] during follow-up. Adjustment for bisphosphonate use was not performed, patients were treated with tamoxifen instead of an aromatase inhibitor, and importantly, no information on BMD measurements was available [15]. Also a sub-study of the AZURE trial found the bone turnover markers P1NP, CTX, and 1-CTP to show good prognostic ability for bone-specific recurrence [16]. None of the markers were prognostic for overall distant recurrence and not predictive of treatment benefit from zoledronic acid. A cohort study showed that pre- breast cancer osteoporosis was not associated with risk of developing bone metastasis [17]. Nevertheless, if patients with untreated pre-cancer osteoporosis developed bone metastases, it occurred approximately 1 year earlier than those without pre-cancer osteoporosis (median time, 1.78 years vs 2.87 years; *p* < 0.001). The fourth study, the MA.27 trial, examined the effects of self-reported osteoporosis and osteoporosis therapy on breast cancer outcomes. The study included 7576 postmenopausal patients during adjuvant aromatase inhibitor treatment (anastrozole/exemestane) for breast cancer [18]. Of patients who did not receive bisphosphonates, the event-free survival rate was 86% (95% CI 78–91%) in case of osteoporosis (*n* = 193) and 87% (95% CI 86–89%) in patients without osteoporosis (*n* = 4672) (no HR reported). While their findings are in line with the results of the current study, it should be recognised that in the MA.27 trial no detailed information was collected on BMD assessments and information on osteoporosis was self-reported [18]. Therefore, current evidence suggests that bone resorption markers might be much more valuable in predicting bone-specific recurrences than BMD.

A possible explanation for not finding an association between a reduced BMD and a lower breast cancer recurrence risk might be that women with osteoporosis have less benefit of aromatase inhibitors because their oestrogen levels tend to be lower than in women with a normal BMD. Hence, in patients with lower intrinsic oestrogen levels—resulting in higher risk of osteoporosis—the breast recurrence risk is reduced in a similar way as in patients with intrinsic higher oestrogen levels treated with aromatase inhibitors.

Earlier studies found bisphosphonates to be valuable in both breast cancer prevention and improved breast cancer survival (when given as adjuvant therapy) irrespective of bisphosphonate type [1–3, 7, 19]. Further, another trial showed that cessation of bisphosphonate treatment after breast cancer diagnosis doubled the risk of developing bone metastases (HR = 2.03, 95% CI 1.26 to 3.26), whereas taking bisphosphonates post-breast cancer diagnosis only, or continuing post-diagnosis reduced the risk of bone metastases (45% and 28% relative reduction, respectively) after a median 5-year follow-up [20]. Also in the MA.27 trial a 5-year absolute 3% improvement of the event-free survival was observed for the patients receiving osteoporosis therapy in comparison with the patients who did not receive osteoporosis therapy [86% versus 89%, HR 0.63 (95% CI 0.40–1.00)] during adjuvant aromatase inhibitor therapy for breast cancer [18]. The benefit was larger in patients without osteoporosis [87% versus 92%, HR 0.65 (95% CI 0.61–0.68)]. The bisphosphonate treatment in our study was also predominantly started for a reduced BMD; however, we did not observe an effect on DRFS. A possible explanation for the diverging observations is that in the MA.27 trial a standard Cox regression analysis was used, potentially overestimating the treatment effect of osteoporosis therapy by introducing ‘immortal time bias’ [21]. When the start of osteoporosis therapy was used as a time dependent covariate the effect on DRFS was not found [18]. Therefore it could be possible that adjuvant bisphosphonate treatment is most effective in patients without osteoporosis. Another explanation for not finding a relationship between bisphosphonate use and late DRFS is the use of the oral bisphosphonates alendronate and risedronate in 86% of the patients using bisphosphonates [12]. The EBCTCG meta-analysis on adjuvant use of bisphosphonates concluded there was insufficient evidence for the use of alendronate and risedronate in the adjuvant setting of breast cancer [7]. Nevertheless, non-prospective trials suggest this relationship also exists for these oral bisphosphonates [22, 23]. Hopefully, future prospective trials will clarify this matter.

Bisphosphonates inhibit osteoclastic bone resorption by attaching to bony surfaces undergoing active resorption and prevent osteocyte and osteoblast apoptosis [24, 25]. Through these mechanisms bisphosphonates increase the BMD, decrease the incidence of osteoporotic fractures, and were implemented as therapy for metastatic skeletal disease [26]. However, several in vitro studies found that bisphosphonates might not only target the osteoclast, but also have direct anti-tumour activity and work synergistic with cytotoxic therapies [27–29]. These might be the mechanisms responsible for the observed clinical benefits in trials investigating the efficacy of zoledronic acid in combination with standard anticancer therapy in early breast cancer patients [2, 4, 5]. Noticeably the indirect metastasis-preventing effect of bisphosphonates seems limited to postmenopausal patients [7], which implies that the effect of oestrogen on the bone microenvironment might play an important role in the benefit from adjuvant bisphosphonate therapy [30].

More recently, the effect of the anti–receptor activator of nuclear factor kappa-B ligand denosumab on breast cancer survival was investigated, showing contrasting results [31, 32]. The ABCSG-18 trial observed a clear advantage of denosumab (60 mg 6-monthly) on disease-free survival (DFS) (HR 0.82, *p* = 0.026) in a study population of postmenopausal women using AIs, who were generally at a low risk of recurrence (25% received prior chemotherapy) [32]. In the denosumab group, DFS was 80.6% at 8 years of follow-up, compared with 77.5% in the placebo group. The D-CARE trial, including both pre- and postmenopausal women of whom 96% received prior chemotherapy, observed no advantage of a more intense regimen of adjuvant denosumab (120 mg monthly for 6 months followed by 120 mg 3-monthly) on DFS (HR 1.04, *p* = 0.57) [31]. A subgroup analysis neither showed an effect for the subgroup of postmenopausal women [31]. Because of these contrasting results denosumab has not been registered (yet) as adjuvant treatment in women with breast cancer. In terms of adverse events, osteonecrosis in the jaw was reported significantly more often in the denosumab arm in the D-CARE trial as compared to placebo [31]. The ABCSG-18 trial, using a lower dose of denosumab, did not observe any differences in the occurrence of osteonecrosis between the use of adjuvant denosumab and placebo [32]. Further, the Medicines and Healthcare products Regulatory Agency recently cautioned that denosumab has been associated with an increased incidence of new primary malignancies (1-year cumulative incidence 1.1%) [33].

Even though this was a planned side-study of the DATA trial, the execution of DEXA scans was not protocolized but was advised to adhere to (inter)national guidelines. This probably explains the absence of BMD measurements within 3 years after randomisation in about one third of the patients. Furthermore, 19.1% of the women with osteoporosis did not use any bisphosphonates which was not in accordance with the recommendations in the national guideline. Additionally, the use of bisphosphonates was not randomised but based on the outcome of the DEXA scans, therefore confounding by indication could not be ruled out. The occurrence of bone metastases was not registered as specific item anymore after the occurrence of distant metastases elsewhere, and therefore we could not use bone metastases free survival as an outcome. Also, with longer follow-up results may change. Nevertheless, this is the first prospective trial studying the relationship between BMD and DRFS with detailed information on BMD in 1142 patients.

In conclusion, we observed no association between BMD and late DRFS in this pre-planned DATA sub-study. Neither did we observe a relationship between bisphosphonate use for a decreased BMD and late DRFS.

## Electronic supplementary material

Below is the link to the electronic supplementary material.

Supplementary file1 (DOCX 16 kb)

Supplementary file2 (PDF 34 kb)
