# Supplementary material for: Breast cancer outcome in relation to bone mineral density and bisphosphonate use: a sub-study of the DATA trial
Source: Breast Cancer Res Treat. 2020 Mar 2;180(3):675–85. doi: 10.1007/s10549-020-05567-9 (PMC7103013; doi:10.1007/s10549-020-05567-9)
Supplement: Supplementary file 1 — Supplementary file1 (DOCX 16 kb) [file 10549_2020_5567_MOESM1_ESM.docx]

| **Supplemental Table 1.** Baseline characteristics of all eligible randomised patients in the DATA study comparing 3 and 6 years of anastrozole after 2 to 3 years of tamoxifen (n=1860) | | | | | | | |
| --- | --- | --- | --- | --- | --- | --- | --- |
|  | **Characteristic** | | | **Total group**  **n = 1860 (%)** | **6 years**  **Anastrozole**  **n = 931 (%)** | **3 years**  **Anastrozole**  **n = 929 (%)** |  |
|  |  | | |  |  |  |  |
|  |  | Age at randomization (years) – no. (%) | |  |  |  |  |
|  |  |  | Median age at randomisation (IQR) |  | 58.1 (51.9; 64.8) | 57.8 (51.5; 64.6) |  |
|  |  |  | < 49 years | 328 (17.6) | 159 (17.0) | 169 (18.2) |  |
|  |  |  | 50-59 years | 735 (39.5) | 372 (40.0) | 363 (39.1) |  |
|  |  |  | ≥ 60 years | 797 (42.9) | 400 (43.0) | 397 (42.7) |  |
|  |  | Tumour status – no. (%) | |  |  |  |  |
|  |  |  | pT1 | 832 (44.8) | 411 (44.2) | 421 (45.4) |  |
|  |  |  | pT2 | 878 (47.3) | 448 (48.2) | 430 (46.3) |  |
|  |  |  | pT3/4 | 148 (8.0) | 71 (7.6) | 77 (8.3) |  |
|  |  |  | TX | 2 | 1 | 1 |  |
|  |  | Nodal status – no. (%) | |  |  |  |  |
|  |  |  | pN0 / pN0(i+) | 605 (32.5) | 295 (31.7) | 310 (33.4) |  |
|  |  |  | pN1 | 991 (53.3) | 490 (52.6) | 501 (53.9) |  |
|  |  |  | pN2 / pN3 | 264 (14.2) | 146 (15.7) | 118 (12.7) |  |
|  |  | Histological grade – no. (%) | |  |  |  |  |
|  |  |  | Grade I | 311 (17.2) | 146 (16.2) | 165 (18.3) |  |
|  |  |  | Grade II | 940 (52.1) | 475 (52.8) | 465 (51.4) |  |
|  |  |  | Grade III | 552 (30.6) | 278 (30.9) | 274 (30.3) |  |
|  |  |  | Unknown | 57 | 32 | 25 |  |
|  |  | Hormone-receptor status – no. (%) | |  |  |  |  |
|  |  |  | ER and PR positive | 1396 (75.1) | 698 (75.0) | 698 (75.1) |  |
|  |  |  | ER or PR positive | 464 (25.0) | 233 (25.0) | 231 (24.9) |  |
|  |  | HER2 status – no. (%) | |  |  |  |  |
|  |  |  | Positive | 37 (2.1) | 21 (2.4) | 27 (3.1) |  |
|  |  |  | Negative | 1675 (97.2) | 841 (97.6) | 834 (96.9) |  |
|  |  |  | Unknown | 137 | 69 | 68 |  |
|  |  | Histology – no. (%) | |  |  |  |  |
|  |  |  | Lobular | 338 (18.2) | 177 (19.0) | 161 (17.3) |  |
|  |  |  | Other | 1522 (81.8) | 754 (81.0) | 768 (82.7) |  |
|  |  | Type of breast surgery – no. (%) | |  |  |  |  |
|  |  |  | Breast-conserving surgery | 919 (49.4) | 474 (50.9) | 445 (48.0) |  |
|  |  |  | Mastectomy | 940 (50.6) | 457 (49.1) | 483 (52.0) |  |
|  |  |  | Unknown / Other | 1 | 0 | 1 |  |
|  |  | Type of axillary surgery – no. (%) | |  |  |  |  |
|  |  |  | Sentinel node only | 505 (27.2) | 263 (28.2) | 242 (26.0) |  |
|  |  |  | Sentinel node plus axillary lymph node dissection | 832 (44.7) | 414 (44.5) | 418 (45.0) |  |
|  |  |  | Axillary lymph node dissection | 499 (26.8) | 240 (25.8) | 259 (27.9) |  |
|  |  |  | None | 24 (1.3) | 14 (1.5) | 10 (1.1) |  |
|  |  | Radiotherapy – no. (%) | |  |  |  |  |
|  |  |  | Local | 520 (28.0) | 264 (28.4) | 256 (27.6) |  |
|  |  |  | Local and regional lymph nodes | 682 (36.7) | 355 (38.1) | 327 (35.2) |  |
|  |  |  | Regional lymph nodes | 45 (2.4) | 25 (2.7) | 20 (2.1) |  |
|  |  |  | None/unknown | 613 (33.0) | 287 (30.8) | 326 (35.1) |  |
|  |  | Prior (neo)adjuvant chemotherapy – no. (%)* | |  |  |  |  |
|  |  |  | Anthracycline- and taxane-containing regimen | 112 (6.0) | 49 (5.3) | 63 (6.8) |  |
|  |  |  | Anthracycline-containing regimen without taxane | 1115 (59.9) | 566 (60.8) | 549 (59.1) |  |
|  |  |  | Taxane-containing regimen without anthracycline | 7 (0.4) | 4 (0.4) | 3 (0.3) |  |
|  |  |  | Regimen without anthracycline or taxane | 25 (1.3) | 9 (1.0) | 16 (1.7) |  |
|  |  |  | No chemotherapy | 601 (32.3) | 303 (32.5) | 298 (32.1) |  |
|  |  | Prior HER2-targeted therapy – no. (%) | |  |  |  |  |
|  |  |  | Yes | 6 (0.3) | 3 (0.3) | 3 (0.3) |  |
|  |  | Previous duration of tamoxifen – no. (%) | |  |  |  |  |
|  |  |  | Median (IQR) (years) | 2.3 (2.1 – 2.5) | 2.3 (2.1 – 2.5) | 2.3 (2.1 – 2.5) |  |
|  |  | Treatment with bone protective agents at inclusion – no. (%) | |  |  |  |  |
|  |  |  | Bisphosphonates | 445 (23.9) | 241 (25.9) | 204 (22.0) |  |
|  |  |  | Vitamin D and/or Calcium | 168 (9.0) | 89 (9.6) | 79 (8.5) |  |
| TX: size of tumour could not be assessed. ER: Oestrogen Receptor. PR: Progesterone Receptor. HER2: human epidermal growth factor receptor 2.  * All patients received cyclophosphamide-based chemotherapy | | | | | | | |
