# Supplementary figures and images for: Breast cancer outcome in relation to bone mineral density and bisphosphonate use: a sub-study of the DATA trial
Source: Breast Cancer Res Treat. 2020 Mar 2;180(3):675–85. doi: 10.1007/s10549-020-05567-9 (PMC7103013; doi:10.1007/s10549-020-05567-9)

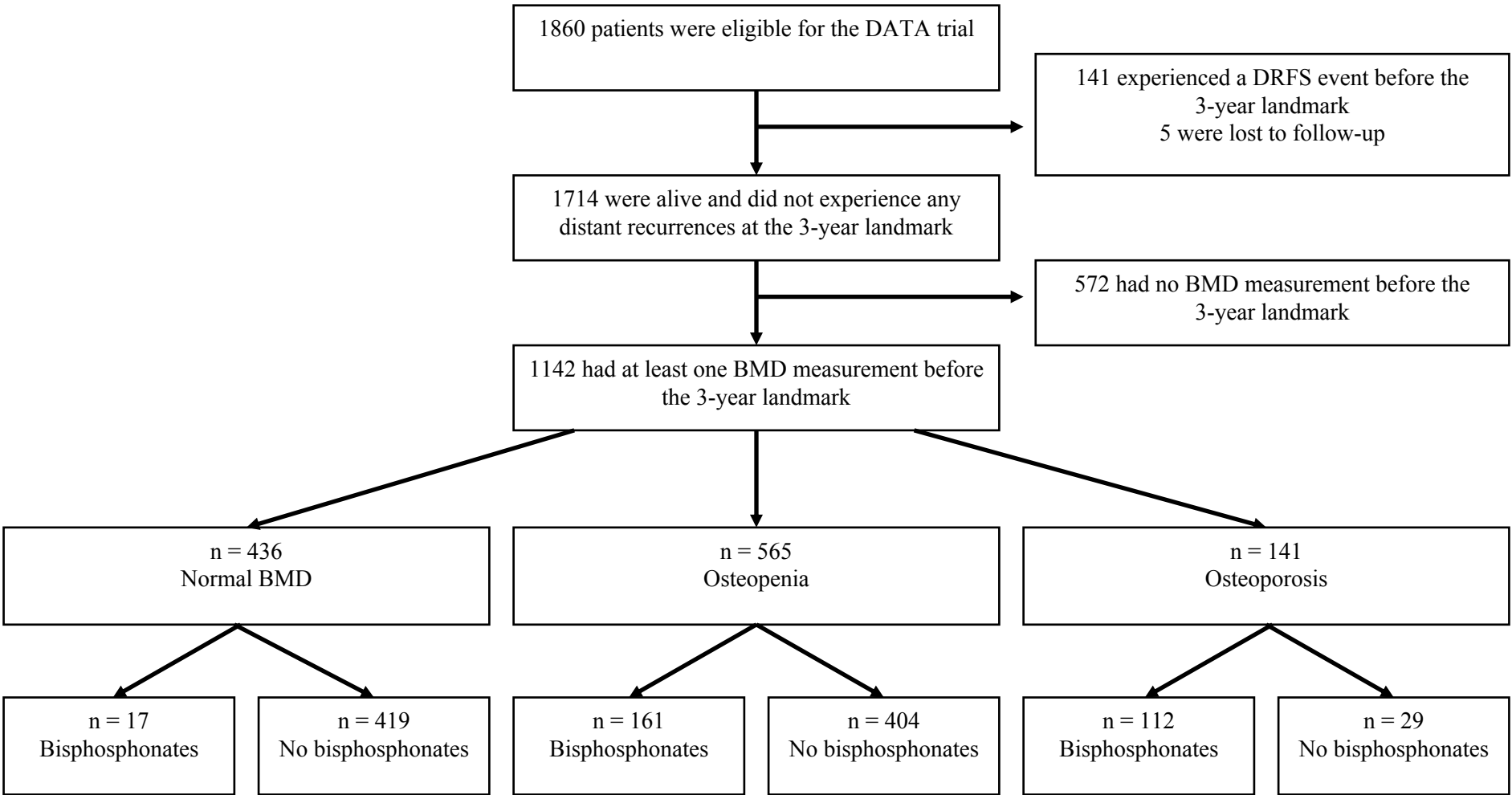

Supplement: Supplementary file 2 — Supplementary file2 (PDF 34 kb) [file 10549_2020_5567_MOESM2_ESM.pdf]
